# Preserving morphology while extracting DNA: a non-destructive field-to-museum protocol for slide-mounted specimens

**DOI:** 10.3897/BDJ.12.e119448

**Published:** 2024-06-07

**Authors:** Santiago Jaume-Schinkel, Björn Müller, Sergio Avila-Calero, Sandra Kukowka, Vera Rduch, Ximo Mengual

**Affiliations:** 1 Leibniz Institute for the Analysis of Biodiversity Change – Museum Koenig, Bonn, Germany Leibniz Institute for the Analysis of Biodiversity Change – Museum Koenig Bonn Germany

**Keywords:** DNA barcoding, Diptera, Psychodidae, moth flies, dark taxa, integrative taxonomy, non-destructive DNA extraction

## Abstract

Our study aimed to develop an optimised laboratory protocol ensuring the preservation of morphological structures and extraction of high-quality DNA sequences from Psychodidae (Insecta, Diptera) specimens. With 310 analysed specimens, we investigated the impact of distinct laboratory treatments by employing two shaking categories (constant and interrupted) with five different incubation periods (16, 12, 8, 4 and 2 hours) during the DNA extraction process. Notably, 80.65% of the specimens exhibited morphological changes during DNA extraction. Our results indicated no statistical difference between constant and interrupted shaking for the total of morphological structures lost. However, within each shaking category, the loss of structures was influenced significantly by the incubation period. Prolonged incubation correlated with increased structural losses, whereas shorter incubation periods caused minor alterations in structures lost. In addition, our results showed a significant difference between constant and interrupted shaking treatments for DNA concentration. Likewise, the incubation period showed differences within each shaking category. Successful COI sequencing was achieved in 89.6% of specimens, with negligible differences in DNA fragment lengths across treatments. Our findings underscore the importance of an optimised protocol and its potential in systematic research involving nematoceran dipteran specimens by balancing morphological integrity and DNA extraction efficiency.

## Introduction

Since the invention of the microscope and Leeuwenhoek's pioneering work with cut specimens in the 17^th^ century, microscopy and slide preparation techniques have evolved significantly. A crucial milestone occurred in the 1830s when slide-mounted specimens, similar to those used today, emerged thanks to the discovery of Canada Balsam as a suitable transparent medium (Bracegirdle 1994), known for its remarkable longevity spanning over a century and a half (Neuhaus et al. 2017).

This historical backdrop sets the stage for understanding the enduring nature of insect slide preparation. Despite the advances in microscopy, the methods for mounting insects on permanent slides have remained surprisingly consistent over the past century. The principles and techniques established in the early to mid-1900s (e.g. Lutz 1922, Gates 1941, Gibbins 2009, Carter et al. 2016) remain relevant today, with only minor adjustments to the pre-mounting process (e.g. using chloral hydrate, potassium or sodium hydroxide, lactic acid for specimen diaphanisation, see Neuhaus et al. (2017)) or the choice of mounting medium (e.g. Berlese fluid, Canada Balsam or Euparal, see Neuhaus et al. (2017)). These methods have proven effective for decades; thus, major changes have been unnecessary.

On the contrary, from the first known classification systems for insects by Aristotle around 350 BC to the modern post-Linneaus and contemporary classifications, the systematical and taxonomical classifications have constantly changed (Engel and Kristensen 2013). With each shift, taxonomists find themselves expanding the character sets to better comprehend the relationships amongst different taxa. This often needs the observation of smaller morphological structures, for which microscope slides become invaluable (Neuhaus et al. 2017).

Furthermore, the advent of DNA Barcoding in 2003 (Hebert et al. 2003) and the integrative taxonomy approaches (Dayrat 2005, Yeates et al. 2010) have called for a harmonisation of both morphological and molecular needs. This involves preserving taxonomically significant structures while extracting DNA for future molecular studies and preserving specimens as voucher specimens for future morphological reference.

In the case of the family Psychodidae (Insecta, Diptera), the traditional techniques for preparing slides have not changed since the 1900s, starting with the Reverend Eaton's preparations around 1900 (Withers 1989). Microscope slides with all sorts of materials can be found in Natural History Collections around the globe. The permanent slide technique (and its variations) is excellent at preserving morphological structures for a very long time, hence the non-necessity of changing them. However, the story changes when we combine this technique with molecular studies. The widely-accepted technique to prepare microscope slides requires a previous maceration of the tissue, removing all traces of DNA in the process (e.g. Ibáñez-Bernal (2005), Curler (2020)).

Our study closely matches the objectives of the German Barcode of Life (GBOL) (https://gbol.bolgermany.de/) initiative and its third phase: “GBOL III: Dark Taxa”. GBOL endeavours to establish a comprehensive DNA barcode reference library for all organisms in Germany, aiming to enhance species identification and contribute to biodiversity research. GBOL III: Dark Taxa specifically targets selected groups of Hymenoptera and Diptera, seeking to elucidate the systematics of highly-diverse and poorly-studied flying insect taxa using genetic information and morphology. By re-evaluating the DNA extraction process and the slide preparation techniques for Psychodidae specimens, our experiment seeks to bridge the gap between traditional slide mounting methods and modern molecular approaches advocated by initiatives such as GBOL III: Dark Taxa.

The aim of the present study is, thus, to develop a new procedure that balances effective DNA extraction with the preservation of crucial diagnostic morphological characteristics for the accurate identification of slide-mounted specimens of moth flies.

## Material and methods

### Specimens

A total of 310 specimens of moth flies (Psychodinae subfamily) were used in this study, collected across different years (2013, 2020 and 2021) using Malaise traps as part of the German Barcode of Life (GBOL) project (Geiger et al. 2016, Hausmann et al. 2020; www.bolgermany.de). Upon collection, all specimens were preserved in 96% ethanol and stored at -20°C until the commencement of the experiment. These specimens exclusively comprised adult moth flies and are presently housed at the Leibniz Institute for the Analysis of Biodiversity Change (LIB) - Museum Koenig Bonn (formerly Zoologisches Forschungsmuseum Alexander Koenig, [ZFMK]), Bonn, Germany. Before DNA extraction, the specimens were identified up to genus level and subsequently added to the GBOL barcoding pipeline.

On average, 10 specimens were selected from each collection year (10 from 2013, 10 from 2020 and 10 from 2021) for each of the experimental treatments and the different incubation periods (see laboratory procedures below), summing 102 specimens for 2013 and 104 specimens for 2020 and also for 2021 (310 total). While efforts were made to evenly distribute these specimens across treatments, collection years and genera, logistical constraints occasionally led to discrepancies in specimen counts. Consequently, in certain instances, eight or thirteen specimens were included from each collection year, based on availability (see Suppl. material 1). Likewise, the majority of the 310 specimens selected were adult males (306 males or 98.7% and four females or 1.29%).

All associated specimen data, including collection years, treatment allocations and genera, are available as supplementary material to enhance the transparency and replicability of our study (see Suppl. materials 1, 2).

### Morphological characters

We created a data matrix to annotate the presence or absence of specific morphological structures before and after laboratory procedures (Suppl. material 2). This allowed us to assess the loss of structures during the DNA extraction process. Each specimen was meticulously examined under a LEICA stereomicroscope model M205 C.

**Figure 1.**
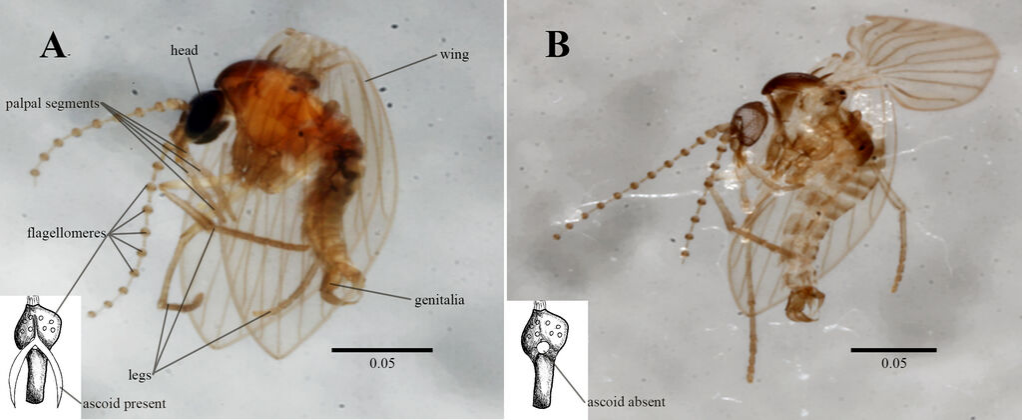
Specimen of the genus *Psychoda* used during DNA extraction. **A.** habitus before extraction; **B.** habitus after DNA extraction, showing how the extraction helps in the diaphanisation of the specimens. Drawing inside a white rectangle shows a flagellomere A) with the ascoid present and B) with the ascoid absent (i.e. lost after DNA extraction). Scales in millimetres.

The examined morphological characters included: the number of antennal segments, the presence of antennal ascoids on each flagellomere (only for those genera with antennal ascoids), the number of palpal segments, the presence of wings, the number of legs and the presence of abdominal terminal segments (genitalia) (see Fig. 1).

The 'total number of changes' refers to the overall count of structural alterations. For instance, if a specimen lost flagellomeres on the left antenna, this was counted as a single change, regardless of the number of flagellomeres lost; if the same specimen lost flagellomeres on the right antenna, this was counted as a separate change, totalling two changes (one for the left antenna and one for the right antenna; see Suppl. material 2). Moreover, if flagellomeres were missing and, as a result, ascoids were lost with them, we counted both separately, as ascoids can be lost without losing flagellomeres. The maximum possible number of changes that could occur in our experiment was 15, including both left and right antennae, ascoids, palpal segments, wings, legs and terminalia. The minimum number of changes was zero.

To minimise structure loss during specimen handling, we exercised great care. Specimens were transferred from Eppendorf vials to watch-glass before morphological examination, using a plastic pipette to avoid direct contact with the specimens. Before and after DNA extractions, specimens were handled with rigid fine-tipped forceps, ensuring that they were held by one wing to minimise potential damage.

### Laboratory procedures (DNA extraction, PCR and PCR conditions)

A non-destructive DNA extraction was performed using the whole body of the specimens at the molecular laboratory of the LIB – Museum Koenig Bonn. Using Qiagen’s magnetic bead-based BioSprint 96 DNA Blood Kit (QIAGEN GmbH - Germany). All specimens were separately lysed in 180 µl ATL buffer and 20 µl Proteinase K in a 12x8 S-Block.

**Figure 2.**
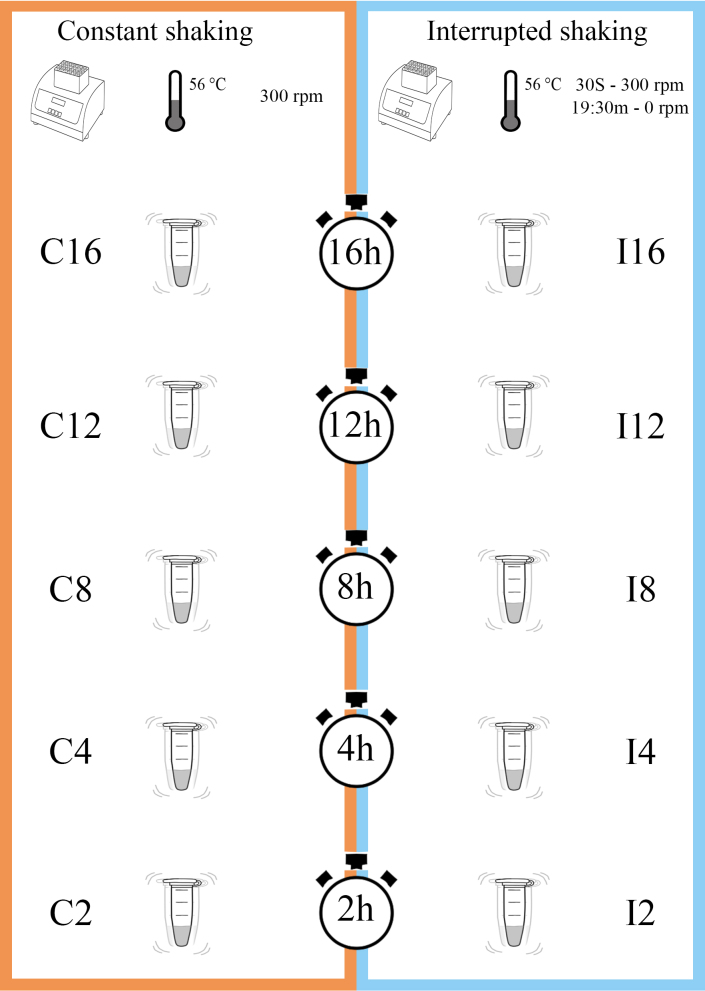
Graphic representation of treatment variables. Constant shaking (C), Interrupted shaking (I). Abbreviations: m = minutes, rpm = revolutions per minute, h = hours, s = seconds.

The incubation was carried out in an Eppendorf Thermomixer® comfort, maintaining a constant temperature of 56°C. Within this set-up, the lysis duration encompassed five distinct timeframes: 16, 12, 8, 4 and 2 hours. Each of these time treatments was associated with either of two lysis methods: 1) Constant shaking (C; treatment categories including incubation periods abbreviated as C16, C12, C8, C4 and C2); or 2) Interrupted shaking (I; treatment categories including incubation periods abbreviated as I16, I12, I8, I4 and I2). The constant shaking treatments were set at 300 rpm; on the contrary, the interrupted shaking treatment consisted of time periods of 20 minutes divided into one cycle of 30 seconds at 300 rpm followed by a cycle of 19.5 minutes at 0 rpm. Accordingly, a total of 10 different treatment groups were used for the experiment. All combined treatment variables can be seen in Fig. 2.

After the incubation period elapsed for each treatment, the animals were removed from the DNA-containing ATL buffer and transferred to 96% ethanol for later morphological examination. The lysate washing, DNA extraction and DNA elution were done with a BioSprint 96 Purification System (Thermo Scientific/ QIAGEN; for more detailed information, see Jafari et al. (2023)).

Using a QIAGEN Multiplex PCR Kit, the master mix for a 96-PCR-well plate is composed of the following: 2000 µl Multiplex, 400 µl Q-Solution, 880 µl RNase-free water and 160 µl of each forward and reverse primer (10 pmol/µl). Each sample well was filled with 36 µl PCR Mastermix and 4 µl from the extracted DNA. The standard primers used in GBOL III for the COI barcode region of insect samples are LCO 1490-JJ (forward) and HCO 2198-JJ (reverse) (Astrin and Stüben 2008). The PCR was carried out with a GeneAmp® PCR System 9700 using a touchdown PCR (TD-PCR) as proposed by Korbie and Mattick (2008). The following conditions were used: initial 15 minutes at 95°C, followed by 94°C denaturation for 35 seconds, 55°C annealing for 90 seconds and 72°C elongation for 90 seconds. Denaturation, annealing and elongation are repeated 15 times, but the annealing temperature was decreased by 1°C in each cycle. After reaching an annealing temperature of 40°C, there was no further reduction in temperature. Denaturation, annealing and elongation are repeated 25 times as described above. A final elongation at 72°C for 10 minutes terminates the PCR and it is then cooled to 12°C permanently.

Bidirectional Sanger sequencing of the COI-PCR products was carried out by BGI BIO Solutions Co, Ltd (Hong Kong, China). Assembly analysis and generating consensus sequence of the sequence data were carried out with Geneious v. 7.1.9 (http://www.geneious.com). The total sequence length was set to 658 bp.

DNA concentration was measured with a Quantus™ Fluorometer (Promega GmbH - Germany) and the QuantiFluor® dsDNA Dye System Kit. Each sample underwent three consecutive measurements, from which the average and median values were calculated. For our analysis involving DNA concentration, we used the average.

Based on our DNA concentration measurements, we selected the 99 samples with the highest DNA concentration for DNA fragment length measurement; for this, each sample was measured with a 5200 Fragment Analyzer System (Agilent Technologies Inc.) using the HS Genomic DNA Kit for Genomic DNA. For the fragment analyser results, we divided the measurements into three groups: fragment lengths from 50 to 500 bp long, 500 to 10,000 bp long and 10,000 to 40,000 bp long. Using the software ProSize 2.0, we obtained a data matrix stating the percentage of fragments that corresponded to each group for each sample.

One observable, but unquantified characteristic was the maceration of soft tissue, as seen in Fig. 1. This outcome was expected during the DNA extraction laboratory procedures, specifically during the lysis process. Tissue maceration is a standard step in the mounting process for Psychodidae and other nematoceran Diptera families, necessary to make internal structures visible for taxonomical identification. Thus, the tissue maceration as a result of the DNA extraction was helpful for the mounting of specimens.

### Preparation of microscope slides

**Figure 3.**
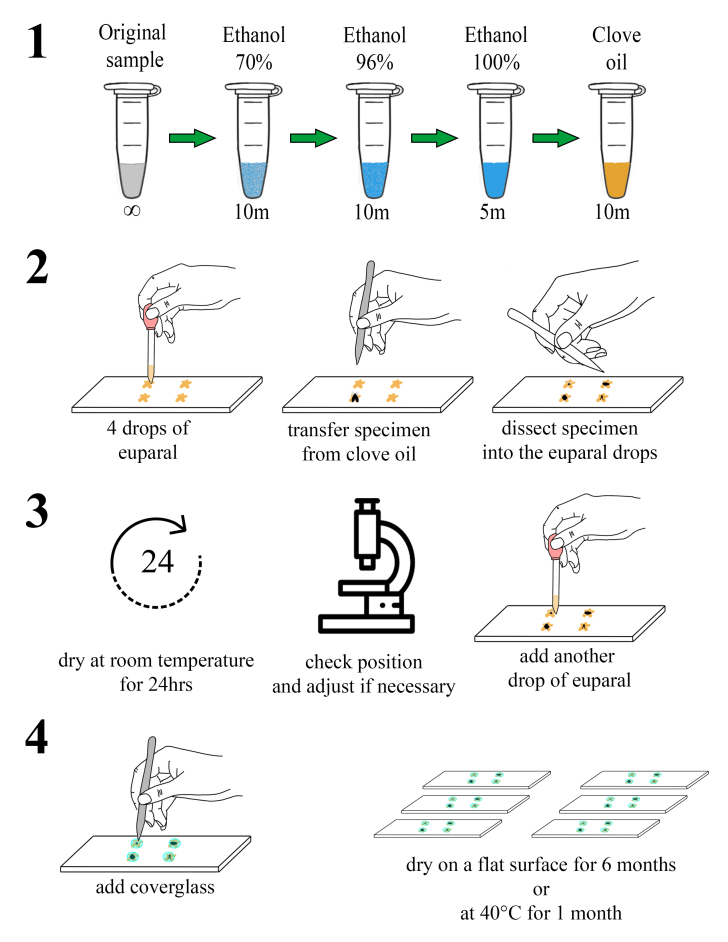
Graphic representation of the slide mounting technique. **1.** dehydration process using different ethanol concentrations and clove oil (m = minutes); **2.** preparation of the microscope slide and dissection of the specimens; **3.** 24-hour lapse for drying the euparal and checking the correct positions of dissected body parts; **4.** Placement cover glasses and drying process.

After the lysis and DNA extraction process, specimens were returned to 96% ethanol and permanent slides were prepared as follows (summarised in Fig. 3):

1. Specimens were rinsed in double-distilled water, followed by a dehydration process in a series of ethanol solutions with different concentrations: first in 70% ethanol for 10 minutes, then in 96% ethanol for another 10 minutes and, finally, in absolute ethanol (100%) for five minutes. Subsequently, the samples were transferred to clove oil and left to soak for at least 10 minutes.
2. Simultaneously, microscope slides were cleaned with 95% ethanol and dried using paper tissue. Four drops (two rows of two drops) of Euparal were distributed in the central part of each microscope slide.
3. Once the dehydration process was complete, specimens were placed in the bottom left drop of Euparal on the microscope slide and dissected directly. The dissected body parts were arranged as follows: wings (or one wing if one was absent) in the top right drop, genitalia in the bottom right drop, head in the top left drop and thorax in the bottom left drop. All dissected body parts were mounted in a dorsal view, except for the thorax and wings, which were positioned laterally. Slides were then left to dry at room temperature for 24 hours.
4. After the 24-hour period, slides were examined under a stereomicroscope to confirm the proper positioning of dissected parts. If everything was correctly placed, another drop of Euparal was applied on top and a 9 mm round cover glass was added. If adjustments were needed, another drop of Euparal was placed on top and, after a 5-10 minutes wait for the new drop to soften the dried Euparal, the body part was repositioned. This process was repeated as necessary until a cover glass was added to each drop of Euparal in the slide.
5. Following the addition of the cover glass, slides were allowed to dry at room temperature for a minimum of six months on a flat surface. Alternatively, this process could be expedited by drying the slides in an incubator set at a constant 40°C for at least 30 days before permanent storage.

### Data analysis

Following the morphological identification of voucher specimens, their DNA sequences underwent comparison with publicly available sequences in The Barcode of Life Data System (commonly known as BOLD; accessible at https://boldsystems.org/) to ensure the absence of cross-contamination during the DNA extraction process.

Statistical analyses were conducted using R (version 2023.03.1+446). The normality of the data distribution was assessed using the Shapiro-Wilk test (Shapiro and Wilk 1965), with a significance threshold set at p = 0.05. Initial comparisons of our dataset for morphological changes and DNA concentration were performed using Kruskal-Wallis tests (Kruskal and Wallis (1952). Subsequently, post-hoc pairwise comparisons were conducted using Dunn's test (Dunn 1964) with Bonferroni correction (Abdi 2010) to identify significant differences between treatment groups. The significance level was set at α = 0.05.

To further explore the data, separate analyses were conducted within constant and interrupted treatment for the different incubation periods. Kruskal-Wallis tests were utilised to compare the DNA concentration and the total morphological changes across the different incubation periods within each treatment. Post-hoc pairwise comparisons were conducted using Dunn's test with Bonferroni correction to identify significant differences between treatment groups. The significance level was set at α = 0.05.

To examine the interaction between DNA fragment percentages, the shaking treatment and the incubation periods, a linear mixed-effects model was fitted using the lme4 package in R (Bates et al. 2015). The model incorporated the random effect of shaking conditions to account for variability between different treatment conditions. Model summaries were generated to assess the significance of the interaction effect and evaluate the influence of shaking conditions on the outcome.

Boxplots were generated to visually represent the data using the R package ggplot2 (Wickham 2016), illustrating the distribution of morphological changes and DNA concentration across different treatment conditions and time points. Additionally, line plots were utilised to display mean and median values, facilitating the interpretation of trends and differences within the dataset.

Additionally, we employed a generalised linear model (GLM) approach to assess the relationship between the response variable "Total changes" and the interaction of the predictor variables "shaking treatments" (constant and interrupted), "incubation periods" and collection year. Likewise, we employed a GLM approach to assess the relationship between DNA concentration as the response variable and the interaction of the same predictor variables. Both GLMs were implemented using the 'glm' function in R with a Poisson and Gaussian family, respectively, to account for count data distribution. The significance of the factors and their interaction was evaluated using the 'Anova' function, facilitating the assessment of the overall model fit and the contribution of each predictor to the variation in the response variable.

## Results

### COI barcoding and species identification

Out of the 310 specimens used for DNA extraction and posterior sequencing, we successfully obtained 278 COI sequences, representing a sequencing success rate of 89.6%. Out of our desired sequence lengh of 658 bp, eight sequences fell short of the desired length (specimen no.: ZFMK-DIP-00097774 [618 bp], ZFMK-DIP-00097780 [624 bp], ZFMK-DIP-00097857 [657 bp], ZFMK-DIP-00097875 [657 bp], ZFMK-DIP-00097881 [631 bp], ZFMK-DIP-00097895 [657 bp], ZFMK-DIP-00097903 [657 bp], ZFMK-DIP-00097906 [608 bp]; see Suppl. material 1 for further information of specimens). Nonetheless, all of the 278 COI sequences obtained are viable to use for species identification. Samples that failed to undergo successful sequencing were slide mounted and their morphological species determination was subsequently performed.

The 310 specimens used for the study belonged to 13 genera and 24 species, namely: *Clytocerus* Eaton, 1904 [*C.ocellaris* (Meigen, 1804)], *Lepiseodina* Enderlein, 1937 [*L.rothschildi* (Eaton, 1913); *L.tristis* (Meigen, 1830)], *Paramormia* Enderlein, 1935 [*P.polyascoidea* (Krek, 1971); *P.ustulata* (Haliday, 1856)], *Pericoma* Haliday, 1856 (*P.blandula* group], *Peripsychoda* Enderlein, 1936 [*P.auriculata* (Haliday, 1839)], *Philosepedon* Eaton, 1904 [*P.humeralis* (Meigen, 1818)], *Pneumia* Enderlein, 1935 [*P.nubila* (Meigen, 1818); *P.trivialis* (Eaton, 1893)], *Psychoda* Latreille, 1797 [*P.alternata* Say, 1824; *P.cinerea* Banks, 1894; *P.gemina* (Eaton,1904); *P.minuta* Banks, 1894; *P.phalaenoides* (Linnaeus, 1758); *Psychodasatchelli* Quate, 1955; *P.* sp., *P.trinodulosa* Tonnoir, 1922], *Seoda* Enderlein, 1935 [*S.ambigua* (Eaton, 1893)], *Telmatoscopus* Eaton, 1904 [*T.advena* (Eaton, 1893)], *Trichomyia* Haliday, 1839 [*T.parvula* Szabó, 1960; *T.urbica* (Haliday in Curtis, 1839)], *Trichopsychoda* Tonnoir, 1922 [*T.hirtella* (Tonnoir, 1919)) and *Ulomyia* Haliday, 1856 [*U.fuliginosa* (Meigen, 1804)]. Specimen data are given in the Suppl. material 1.

It is worth mentioning that some specimens were not identified to species level (e.g. *Pericomablandula* group [8 specimens]; *Psychoda* sp. [1 specimen]), corresponding to 2.90% out of the 310 individuals. This decision was made mainly based on two reasons: 1) the species group requires further examination and a taxonomic revision to properly address the species within and 2) the morphological characters do not fully match the closest related species and no DNA barcode is available for comparison of molecular data. Therefore, to avoid including a species name that later needs amendment, the determination remains at the genus level.

### Data normality

The Shapiro-Wilk normality tests were performed on three variables in our dataset: DNA concentration, total changes in structures lost and DNA fragment measurements. For DNA concentration, the data were not normaly distributed (W = 0.9062, p = 5.848e-13). Similarly, our change in morphological structures is not normally distributed, as the Shapiro-Wilk test yielded a very low p-value (W = 0.9074, p = 7.328e-13). Likewise, the percentage of the DNA fragment measurements are not normally distributed (W = 0.4032, p = 2.2e-16).

### Loss of structures

**Figure 4.**
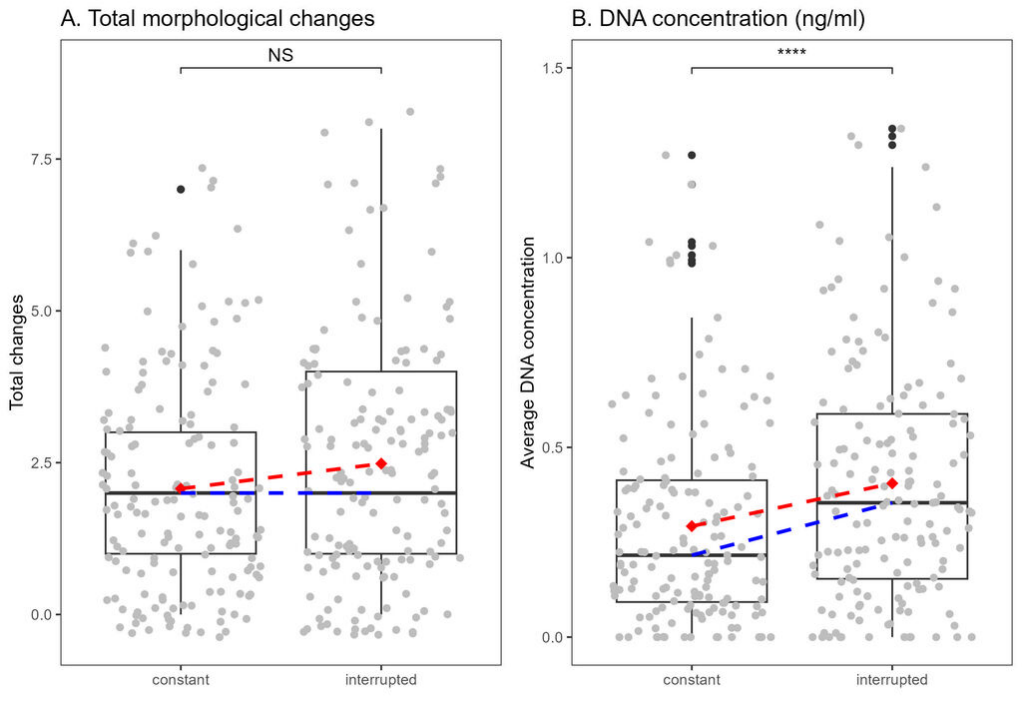
**A**. Total morphological changes occurred during DNA extraction; **B.** DNA concentration (ng/ml) obtained during DNA extraction. Significance values obtained from Kruskal-Wallis test with post-hoc Dunn test, Bonferroni corrected. Values: NS = not significant, **** = p < 0.0001. The Red dashed line indicates the mean and the blue dashed line indicates the median.

Out of the 310 specimens used in our analyses, 60 (19.35%) did not exhibit any changes in the structures lost during the DNA extraction process, while 250 (80.65%) displayed at least one change (see Fig. 4).

Based on the Kruskal-Wallis test, there were no significant differences detected amongst shaking treatments (constant and interrupted) in terms of total morphological changes (χ² = 3.31976, p > 0.05). Therefore, no post-hoc pairwise comparisons were performed. This suggests that the different treatments did not lead to statistically significant variations in total morphological changes amongst the experimental groups.

**Figure 5.**
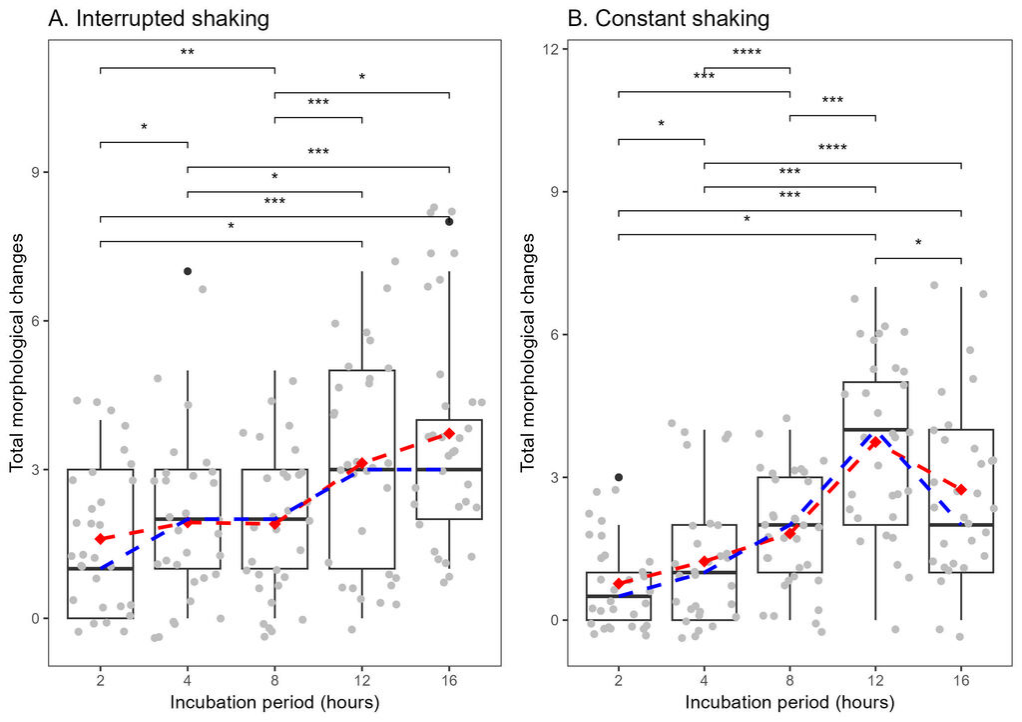
Total morphological structures that were lost within each shaking category. **A.** Interrupted shaking; **B.** Constant shaking. Significance values obtained from Kruskal-Wallis test with post-hoc Dunn test, Bonferroni corrected. Values: * = p < 0.05, ** = p < 0.01, *** = p < 0.001, **** = p < 0.0001. The Red dashed line indicates the mean and the blue dashed line indicates the median.

To evaluate potential differences in the variable 'Total_changes' (loss of morphological strucutures) amongst the groups within the treatment 'constant shaking' (hereafter abbreviated as C, followed by the incubation hours, for example, C2), the Kruskal-Wallis test reported a statistically significant result (χ² = 53.429, df = 4, p < 0.001). Post-hoc Dunn's tests with Bonferroni correction were performed, revealing significant differences between various group pairs. Specifically, C12 showed no significant difference compared to C16 (p = 0.280), but had significantly lower values compared to C2 (p < 0.001) and C4 (p < 0.001). C16 exhibited significantly higher values than C2 (p < 0.001) and C4 (p = 0.003). C2 also had significantly lower values compared to C4 (p < 0.001). C12 did not significantly differ from C8 (p = 0.483), while C16 showed significantly higher values compared to C8 (p = 0.002). No significant differences were observed between C2 and C8 (p = 1) nor between C4 and C8 (p = 0.335) (Fig. 5).

Likewise, when comparing the incubation periods within the interrupted treatment (hereafter abbreviated as I, followed by the incubation hours, for example, I2), the Kruskal-Wallis test yielded a statistically significant result (χ² = 23.301, df = 4, p < 0.001). Post-hoc Dunn's tests with Bonferroni correction revealed significant differences between several group pairs. Notably, I16 displayed significantly lower values compared to I2 (p = 0.002) and I4 (p = 0.0004), while no significant differences were observed between I16 and I12 (p = 0.151). Additionally, I2 exhibited significantly higher values than I4 (p = 0.004). These findings suggest that the total loss of morphological structures varies significantly across different groups, based on the incubation period (Fig. 5).

Our GLM analysis revealed significant effects of the shaking treatment (χ² = 5.240, df = 1, p = 0.02207) and the incubation period (χ² = 96.760, df = 1, p < 2e-16) on the total changes, indicating that both factors independently influence the outcome variable. Specifically, interrupted shaking was associated with a significant increase in the total changes (Estimate = 0.422, Std. Error = 0.1749, z = 2.413, p = 0.0158), while an increase in the incubation period was also linked with increased morphological structures lost (Estimate = 0.08573, Std. Error = 0.01126, z = 7.610, p < 2.74e-14). However, the interaction between shaking and incubation period did not reach statistical significance (χ² = 2.521, df = 1, p = 0.11232), suggesting that the combined effect of these factors may not be different from their individual effects. Overall, the model provided a good fit to the data, as evidenced by the relatively low residual deviance (447.09 on 306 degrees of freedom) and the corresponding Akaike Information Criterion (AIC) value of 1148.

### DNA concentration

The DNA concentration exhibited a range from 0.00 to 1.33 ng/µl, with a mean value of 0.32 ng/µl, a median value of 0.25 ng/µl and a mode of 0.00 ng/µl. Remarkably, even in cases where the concentration was measured as 0.00 ng/µl, we were able to obtain high-quality COI barcodes of the desired length (658 bp).

The Kruskal-Wallis test was conducted to compare DNA concentrations between constant and interrupted treatments. The test revealed a statistically significant difference in DNA concentration between the two treatment groups (χ² = 10.92187, df = 1, p < 0.05). Post-hoc pairwise comparisons using Dunn's test with Bonferroni correction further elucidated the findings. The interrupted treatment group exhibited a significantly higher mean DNA concentration (mean = 0.405) compared to the constant treatment group (mean = 0.292), with a p-value of 0.0005 (see Fig. 4).

Within the constant shaking treatment, the Kruskal-Wallis test found no significant differences in the average DNA concentration amongst the different incubation periods (χ² = 5.5167, df = 1, p-value > 0.05). Therefore, post-hoc pairwise comparisons using Dunn's test were not performed.

**Figure 6.**
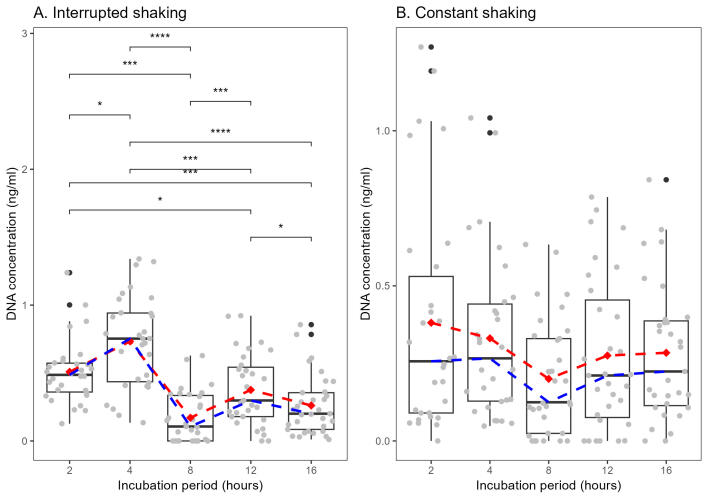
DNA concentration within each shaking category. **A.** Interrupted shaking. **B.** Constant shaking. Significance values obtained from Kruskal-Wallis test with post-hoc Dunn test, Bonferroni corrected. Values: NS = not significant, * = p < 0.05, ** = p < 0.01, *** = p < 0.001, **** = p < 0.0001. The Red dashed line indicates the mean and the blue dashed line indicates the median.

Conversely, the Kruskal-Wallis test revealed a statistically significant difference in average DNA concentration amongst the different incubation periods within the 'interrupted' treatment (χ² = 58.657, df = 4, p < 0.001). Post-hoc pairwise comparisons using Dunn's test with Bonferroni correction further elucidated significant differences between certain group pairs. Notably, I2 exhibited significantly lower average DNA concentration compared to I16 (p = 0.027) and I4 (p = 0.0002). Additionally, I4 displayed significantly lower average DNA concentration compared to I8 (p < 0.001). No significant differences were detected between I12 and other incubation periods. (Fig. 6).

### DNA fragments

The Shapiro-Wilk test revealed that the DNA fragment measurements were not normally distributed (W = 0.40322, p-value < 2.2e-16) (Suppl. material 3). The linear mixed effects model indicated that the incubation period had a non-significant effect on the response (Estimate = 116.3, Std. Error = 157.5, t-value = 0.738, p > 0.05). Random effects analysis revealed substantial variability between different levels of shaking (constant and interrupted), with a significant variance of 538440 (Std. Dev. = 733.8). The model provided a good fit to the data, as indicated by the REML criterion at convergence (2032.8).

### Collection year

The results of our GLM show that the collection year variable on both total changes and the DNA concentration is significant. In the case of total changes, the GLM demonstrated a marginally significant effect (p = 0.084). The negative coefficient estimate (-0.11728) suggests a potential decreasing trend in the total changes for recent collection years. In other words, specimens that have been collected recently (2020 and 2021) lost fewer structures compared to those collected in 2013. Conversely, when examining DNA concentration, the main effect of the collection year was significant (p = 0.041). Although the coefficients for individual years were not statistically significant, the overall effect suggests a potential difference in DNA concentration across different collection years.

## Discussion

### Loss of structures

Fragile specimens, such as Psychodidae and other nematoceran flies, commonly sustain damage, including the loss of antennae, legs and wings, during mass-collecting methods such as CDC traps, Malaise traps or yellow pan traps. Despite the meticulous handling and the precautions taken during transportation and storage, specimen damage remains a persistent issue (Karlsson et al. 2020). Some dipterists prefer hand-netting to mitigate these issues, as up to 50% of specimens in large samples may not be suitable for morphological identification (Jaschhof and Jaschhof 2009).

Moreover, many samples stored for extended periods may not receive proper maintenance or monitoring. Ethanol concentrations may become inadequate for effective specimen preservation and bulk samples are often stored at room temperature, leading to the deterioration of both specimens and their DNA. Recent projects, such as The Swedish Malaise Trap Project (Karlsson et al. 2020) and the third phase of the German Barcode of Life initiative, GBOL III: Dark Taxa (Hausmann et al. 2020), aim to address these challenges. However, managing large volumes of sampled material remains a persistent issue. Therefore, emphasising proper management is crucial to ensure the preservation of both DNA and morphological structures over time.

During our study, we found that the majority of moth fly specimens retrieved from Malaise traps exhibit evident structural changes, such as the loss of ascoids, flagellomeres and legs preior to our morphological examination. Despite our efforts to minimise handling-induced damage, 80.65% of the specimens displayed further alterations in morphological structures during the experiment. Despite the loss of morphological structures, we successfully identified to species level 95% of the 310 specimens analysed.

Our results show that there is no significant statistical difference between shaking treatments (constant and interrupted) regarding the loss of morphological structures. However, when evaluating the incubation periods within each treatment, we found statistically significant differences. Within the constant treatment, C2 had the lowest number of structures lost (see Fig. 5), while C12 presented the highest number, although C12 did not differ from C8. Likewise, within the interrupted treatment, there is a trend suggesting that shorter incubation periods result in fewer morphological structures lost and longer incubation periods result in more structures lost (Fig. 5).

Our findings suggest that varying the incubation period during DNA extraction directly influences the loss of morphological structures. It is important to note the significance of this observation, especially for genera such as *Psychoda*, where terminal flagellomeres are often used for species identification. Likewise, morphological characters found in the antennae (e.g. the shape of antennal segments, the presence, arrangement and number of ascoids) are often used for genus-level determination. With each structure lost during collection, storage and specimen handling, we lose valuable diagnostic features, especially when studying hyper-diverse taxa, such as moth flies.

While meticulous handling and storage precautions were undertaken to mitigate damage to the specimens, our statistical analysis reveals a significant effect of the collection year on the total changes (p = 0.084). This finding suggests a trend of decreasing morphological changes in recent collection years. Such a trend underscores the importance of understanding the dynamics of specimen damage through time and the need for continued attention to specimen management practices over time.

### DNA concentration

Some of the specimens used in our study presented an average DNA concentration of 0.0 ng/µl. Surprisingly, this minimal concentration did not hinder the successful generation of high-quality COI sequences. It is important to clarify that, according to the QuantiFluor® user manual, concentrations below the lower limit of 0.01 ng/µl are not displayed during measurement, resulting in registered values of zero. Therefore, a reading of zero does not imply a lack of DNA extracted, but rather a concentration below the machine's detectable threshold.

Our results indicate that there is a significant statistical difference in the average DNA concentration between treatments (constant and interrupted shaking). The interrupted treatment exhibited a higher mean DNA concentration compared to the constant treatment (Fig. 4). Specifically, within the interrupted shaking, I4 yielded the highest average DNA concentration (see Fig. 6). On the contrary, C8 and I8 yielded the lowest average DNA concentration (see Figs 5, 6, respectively). Overall, our findings suggest that interrupted shaking during different incubation periods yields higher average DNA concentrations compared to constant shaking. Nonetheless, continuous shaking shows a trend in lower DNA concentrations, but fewer losses in morphological structures. Conversely, interrupted shaking leads to higher average DNA concentrations and a higher loss of morphological structures. This discrepancy raises intriguing questions regarding the impact of shaking intervals on DNA concentration across different treatments, warranting further investigation.

Considering the minuscule size of the average adult moth fly (less than 5 millimetres in total body length) and the natural degradation of DNA in preserved specimens, the observed low DNA concentration is not unexpected. Based on a quick exploration of our data, we observed that the genera *Seoda* and *Telmatoscopus* exhibited a notably higher DNA concentration when compared to other genera included in our sampling. This variance may be attributed to the larger body size of species within the *Seoda* and *Telmatoscopus* genera compared to other genera. However, our study's scope did not allow for an in-depth exploration of the specific relationship between body size and DNA concentration and further research is needed to explore the relationship between body size and DNA concentration.

Furthermore, it is crucial to distinguish between DNA concentrations measured directly for our study and the COI sequences obtained from the PCR product sent to BGI. The PCR product amplifies the DNA from the sample, enhancing the potential for obtaining high-quality COI sequences that are not necesarilly dependent on the DNA concentration obtained from our samples.

The Gaussian GLM analysis revealed significant effects of the shaking treatments (t = 3.966, p = 9.10e-05), especially for the interrupted:incubation period interaction (t = -2.129, p = 0.034) and for the incubation period (t = -1.549, p = 0.122) on the DNA concentration. The intercept term was also significant (t = -7.559, p = 4.75e-13). Specifically, interrupted shaking was associated with a significant increase in DNA concentration (Estimate = 0.64103, Std. Error = 0.16162, p < 0.001), while the interaction between interrupted shaking and incubation period demonstrated a significant negative effect (Estimate = -0.04258, Std. Error = 0.02000, p = 0.034). Additionally, a decrease in the incubation period was marginally associated with a decrease in DNA concentration (Estimate = -0.02415, Std. Error = 0.01559, p = 0.122). The model exhibited a good fit to the data, as evidenced by the low residual deviance (23.807 on 306 degrees of freedom) and the corresponding Akaike Information Criterion (AIC) value of 94.095. Analysis of deviance further confirmed the significance of the shaking treatment (χ² = 17.748, df = 1, p = 2.522e-05), incubation period (χ² = 31.684, df = 1, p = 1.814e-08) and their interaction (χ² = 3.930, df = 1, p = 0.04743) on DNA concentration.

Our analysis revealed a marginal, yet significant effect of the collection year variable on the DNA concentration (p = 0.041). Although individual collection years were not statistically significant, the overall effect suggests a potential difference in DNA concentration across different years. These findings suggest a temporal dynamic that could influence DNA concentration and morphological integrity, underscoring the complexity of factors affecting specimen preservation and DNA extraction efficiency.

### General discussion

Our results indicate that groups C2, C4, I2 and I4 were the optimal choices when considering both the number of structures lost and the DNA concentration in our samples (Figs 5, 6). Particularly noteworthy is I4, which demonstrated a relatively low number of lost structures (Fig. 5) coupled with one of the highest DNA concentrations (Fig. 6). Similarly, treatments C4 and C2 also exhibited favourable DNA quantity (Fig. 6), with the level of structural loss remaining on the lower end of the spectrum compared to other categories.

C2 presented the lowest number of morphological structures lost (see Fig. 5), but yielded less DNA than other variables (Fig. 6). However, it is important to highlight that, despite the lower DNA median concentration in C2 compared to others, we successfully obtained high-quality COI sequences from 29 out of 30 specimens within this treatment group. This suggests that, while the DNA yield might be lower, C2 has an impressive capability to preserve morphological structures, indicating a favourable equilibrium of providing adequate COI sequences whithout compromising the morphological structures of the specimens.

The results of the generalised linear modeling (GLM) analyses shed light on the factors influencing the observed changes in the total changes and DNA concentration. In the case of total changes, our findings indicate significant effects of both shaking and incubation periods, suggesting that interrupted shaking and the incubation period independently impact the total number of changes observed. Interestingly, while the interaction between these factors did not reach statistical significance, their individual effects remain noteworthy. Conversely, for the DNA concentration, the GLM revealed significant effects observed for shaking, incubation period and their interaction. Interrupted shaking was associated with an increase in DNA concentration, while the interaction effect between the shaking treatment and the incubation period demonstrated a significant negative association with DNA concentration, suggesting a potential moderating effect. Future research could explore additional factors that may influence these outcomes and further elucidate the underlying mechanisms driving these effects.

Overall, our findings suggest that C2 strikes a delicate balance between minimising structural loss and yielding viable DNA sequences. This observation underscores its potential efficacy in preserving morphological integrity, while still ensuring an adequate yield of DNA for downstream analysis. Consequently, a DNA extraction protocol involving constant shaking with a 2-hour incubation period emerges as a promising choice for future studies involving DNA extraction in mothflies.

The selection of a 2-hour incubation period offers several advantages. Firstly, it promises a relatively short processing time for each set of samples, facilitating efficient throughput in laboratory workflows. With the potential to process multiple sets of samples in a single day, researchers can significantly expedite data collection and analysis compared to protocols requiring longer incubation periods, such as those exceeding 8 hours.

Moreover, the balance achieved by C2 suggests that it may be particularly well-suited for applications where preserving morphological features alongside DNA integrity is crucial. This is especially pertinent in studies involving delicate or rare specimens, where maintaining structural integrity is paramount for accurate taxonomic identification or morphological analysis.

### Notes on mounting media

This study used Euparal as mounting media for the microscope slides as it works well, although some researchers prefer Canada Balsam or other mounting media (see Neuhaus et al. (2017)). Canada Balsam is also suitable to be used after DNA extraction. The choice of mounting medium is often a matter of personal preference, costs and availability. At present, both media (Euparal and Canada Balsam) are widely used amongst taxonomists and both have proven to be effective for the long-term preservation of specimens (more than 50 and 150 years, respectively) (Neuhaus et al. 2017).

During the process of preparing specimens on microscope slides, the application of mounting medium can vary in quantity, typically ranging from one to several drops, with five or six drops often considered the maximum. This variability is contingent upon several factors, including the size of the specimen, the dimensions of the cover glass, the availability of cover glass pieces and the number of dissected structures per specimen. For our study, we used four small round cover glasses, each with a diameter of 9 mm, for the dissected structures (genitalia, head, thorax and wings). However, it is common to encounter microscope slides in entomological collections where the entire specimen is covered by a single 12 mm round cover glass or by a square or rectangular cover glass. Researchers frequently employ the practice of cutting square or rectangular cover glasses into smaller pieces, utilising each fragment to cover dissected structures of the specimens.

Steps 3 and 4 detailed in our proposed method (also see Fig. 3), which pertain to the slide preparation process, can be readily adapted to accommodate the specific requirements and available materials of individual researchers. This adaptability ensures flexibility in the mounting process, allowing researchers to tailor their approach, based on the studied taxa, as well as the resources at their disposal.

## Conclusions

In summary, the persistent challenges surrounding specimen damage and DNA preservation in entomological studies, particularly amongst fragile species like Psychodidae and other nematoceran families, highlight the need for effective strategies. Our findings suggest that using different shaking treatments across shorter incubation periods during DNA extraction hold promise in mitigating structural losses while maintaining good DNA yield.

This research underscores the delicate balance between preserving morphological integrity and obtaining viable COI DNA barcodes. Constant shaking with an incubation period of two hours (C2) demonstrates potential efficacy in preserving structures while providing adequate DNA for analysis. Nonetheless, the loss of even minor morphological structures, during sample handling and post-extraction morphological identification, in poorly-studied taxa raises concerns about the potential loss of diagnostic information. Therefore, handling specimens with care during collection, DNA extraction and slide preparation is crucial to avoid missing valuable morphological characters.

The ongoing refinement of methodologies in taxonomical studies remains essential in addressing the enduring challenges of specimen preservation and molecular data analysis. This imperative stems from the continuous evolution of new methods and technologies, especially with next generation sequencing techniques, emphasising the need for adaptability and innovation to effectively overcome upcoming challenges.

The implications of these findings extend beyond the realm of moth-fly research, offering insights into the optimisation of DNA extraction protocols across diverse taxa and sample types. By prioritising both structural preservation and DNA yield, protocols like ours hold promise for enhancing the efficiency and reliability of genetic studies, ultimately advancing our understanding of biological diversity and evolutionary processes. Further investigation and validation across different organisms and experimental conditions will be essential to fully harness the potential of such protocols in molecular research.

## Supplementary Material

DC1FB02C-2E11-5B73-B8D0-B56D5C19CD02 10.3897/BDJ.12.e119448.suppl1 Supplementary material 1 Specimen Collection Data Data type collection data Brief description Collection data for all specimens used in the study. File: oo_1049047.xlsx https://binary.pensoft.net/file/1049047 Jaume-Schinkel, S. et al

5727CE4C-8420-5E17-B364-9B01EE8D38BD 10.3897/BDJ.12.e119448.suppl2 Supplementary material 2 Experiment data Data type numerical Brief description Data used for statistical analysis. File: oo_1049048.csv https://binary.pensoft.net/file/1049048 Jaume-Schinkel, S. et al

371516EE-6F29-5EB6-9723-4E0560F83298 10.3897/BDJ.12.e119448.suppl3 Supplementary material 3 Fragment Analyser Data Data type Numerical Brief description Data obtained from the fragment analyser in percentage of fragment lengths. File: oo_1049050.csv https://binary.pensoft.net/file/1049050 Jaume-Schinkel, S. et al
